# Amebic Liver Abscess Complicated With a Pleural Effusion: A Case Report

**DOI:** 10.7759/cureus.30126

**Published:** 2022-10-10

**Authors:** Maria S Salazar, Carlos D Maya, Mario Cervantes, Ajmani Surainder

**Affiliations:** 1 Pathology and Laboratory Medicine, Universidad de Oriente, Ciudad Bolivar, VEN; 2 Pathology and Laboratory Medicine, HCA Houston Healthcare West, Houston, USA; 3 Pathology and Laboratory Medicine, Universidad Xochicalco (Campus Mexicali), Ensenada, MEX; 4 Internal Medicine, HCA Houston Healthcare West, Houston, USA

**Keywords:** metronidazole, antibody amebic test, pleural effusion, amebic liver abscess, entamoeba histolytica, amebiasis

## Abstract

Amebiasis is a fecal-oral transmitted parasitic infection caused by the protozoan *Entamoeba histolytica,* and is generally seen in migrants and travelers of endemic areas. Extraintestinal infection often involves the liver, causing amebic liver abscesses. Twenty to thirty percent of these patients have pleuropulmonary involvement as a complication. The diagnosis is based on clinical, imaging, and serology studies.

A 35-year-old male from New Guinea presented to the emergency department with right upper quadrant pain that radiates to the right shoulder, epigastric pain, and fever. Laboratory results showed an increase in hepatic enzymes; days later leukocytosis was reported. Ultrasound revealed hepatomegaly with heterogeneous masses, and three complex cystic hepatic abscesses were found on a CT scan. Percutaneous drainage was placed. Chest X-ray showed bilateral pleural effusion that required a thoracentesis days after. A pigtail catheter was placed. Three amebic antibody tests were performed with a negative result for the first time, equivocal on the second time, and a positive result on the last one. Twenty-six days later the patient was discharged.

Amebiasis is a rare and benign condition in the United States, that can cause abdominal cramping, watery diarrhea, and weight loss. A very low percentage of patients will develop an amebic liver abscess, which can be fatal. Amebic liver abscess may rupture and spread to the peritoneum, pleural space, or pericardium. The serum antigen followed by the serology test contributes to the accurate diagnosis. The first antibody amebic test performed on a patient, has a high probability of a false negative result, due to this possibility, the test must be repeated. Metronidazole remains the drug of choice, and therapeutic aspiration is occasionally required as an adjunct to antiparasitic therapy.

## Introduction

Amebiasis is a parasitic infection caused by the protozoan *Entamoeba histolytica* [1]. As a result of fecal-oral transmission, the majority of infections occur in underdeveloped nations, such as the Indian subcontinent, Southeast Asia, sub-Saharan Africa, and Central and South America [2]. When food handlers are shedding cysts or when crops are cultivated in feces-contaminated soil, fertilizer, or water, food-borne exposure is most common and especially likely. Conversely, oral and anal sex, as well as direct rectal inoculation using colonic irrigation devices are less typical ways of transmission [3]. Over 100,000 people die each year from colitis or other extraintestinal diseases that affect over 50 million people in the world. There are four intestinal ameba species that have the same morphological traits; the most symptomatic disease is caused by *E. histolytica* [4]. Ninety percent of infections may be asymptomatic; however, symptomatic infections may cause dysentery or extraintestinal disease [3,5]. Extraintestinal infection by *E. histolytica* most often involves the liver, resulting in amebic liver abscess in around 2-5% of the patients [1,3].

Most of the symptomatic individuals have right upper quadrant pain that may be dull or pleuritic in character and may radiate to the shoulder. In addition, patients are typically febrile. Common symptoms include right-side pleural effusion and point discomfort above the liver. The most common complication of an amebic liver abscess is pleuropulmonary involvement, which is documented in 20-30% of patients. Both percutaneous catheter drainage and medical treatment are necessary for abscesses that rupture into the peritoneum and manifest as either an indolent leak or an acute abdomen [3].

Diagnosis depends on clinical findings, radiographic or ultrasound imaging methods, and serological tests. An amebic liver abscess is considered as a potentially fatal condition for which a prompt diagnosis is essential. Amebic serology is often carried out in reference laboratories, and findings can be obtained in days to weeks [6].

Here, we present a case of an adult from New Guinea that exhibited three complex cystic amebic hepatic abscesses, complicated with a pleural effusion (empyema) that required a thoracentesis.

## Case presentation

A 35-year-old native New Guinea male, with no prior medical history, presented to the Emergency Room exhibiting constant severe stabbing pain in the right upper quadrant, which that radiated to the right shoulder, epigastric pain, and fever for the past five days. This was associated with vomiting, diarrhea, bloating, and one episode of hemoptysis. He denied any previous history of similar complaints. Upon examination, the patient was alert with apparent discomfort. The blood pressure was 104/67 mmHg, pulse rate 117 bpm, and respiratory rate 14 rpm. Abdominal examination revealed a soft, distended right upper quadrant and epigastric pain to palpation, with no guarding/rebound. The patient was managed with pain medication on the first day of admission. Laboratory findings showed an elevation of hepatic enzymes (Table 1).

**Table 1:** Laboratory analyses during the patient’s hospitalization representing the progress of the disease U/L, units per liter; K/mm3, thousands per cubic millimeter; AST, aspartate aminotransferase; ALT, alanine transaminase; ALP, alkaline phosphatase; WBC, white blood cells.

	06/08/22	06/10/22	07/04/22	Reference Value
AST (U/L)	245	122	38	8-33
ALT (U/L)	147	118	48	7-56
Total ALP (U/L)	182	170	93	45-115
WBC (K/mm^3^)	16.7	19.1	3.2	3.3-8.7
Platelet count (K/mm^3^)	44	74	364	150-450

**Figure 1:**
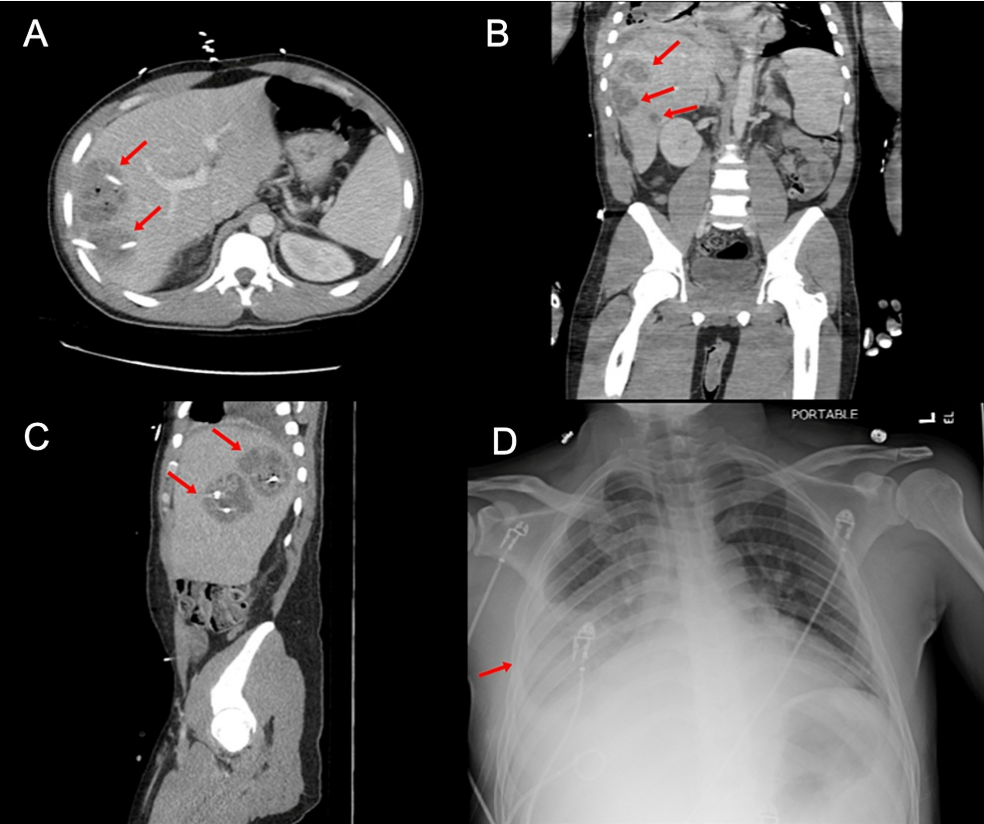
Transverse (A), coronal (B), and sagittal (B) anatomic projections of contrast CT abdomen and pelvis demonstrating three complex cystic hepatic lesions (red arrows); (D) Chest X-ray anteroposterior view, showing interstitial worsening in the aeration of the right lower lobe associated with increase in the right pleural effusion (red arrow)

On abdominal ultrasonography, hepatomegaly measuring 10.5x3 cm and heterogeneous masses within the right hepatic lobe were noted. A CT scan was also performed reporting: three complex cystic hepatic lesions suspicious of abscesses (6x5 cm, 7x5 cm, and 2.5x2 cm respectively) in the right hepatic lobe, hepatic steatosis, and bilateral multifocal ground glass infiltrates consistent with pneumonia (Figure 1). During this procedure the patient became hypotensive. The systolic blood pressure dropped to around 70 mmHg. The patient remained hypotensive after a 3 L bolus. Even though he did not meet the sepsis criteria on initial evaluation, a sepsis bundle was later required, in which 30 cc/kg of IV fluids were given along with broad-spectrum antibiotics (piperacillin/tazobactam 3.3 g IV every eight hours and metronidazole 100 ML IV every eight hours). A chest X-ray suggested interstitial worsening in the aeration in the right lower lobe associated with an increase in right pleural effusion (Figure 1).

On the second day of hospitalization, planned abscess drainage by interventional radiology was in progress. The patient underwent a percutaneous drain placement x2 and percutaneous aspiration x1 procedure; superior hepatic abscess (60 cc), inferior hepatic abscess (30 cc), and medial right hepatic abscess (5 cc), with a sample sent for culture that showed alpha *Streptococcus* infection resulting in a change in the treatment from piperacillin/tazobactam to ampicillin/sulbactam 3 gm IV every six hours. The patient’s symptoms did not improve, exhibiting tachycardia, fever of 100^o^F, right upper quadrant pain, and blood pressure of 148/102 mmHg. The WBC count increased from 4,000 to 19,100 k/mm^3^. Therefore, the dose of metronidazole was increased to 750 mg every eight hours orally. In the meantime, a stool culture (trichrome stain smear) was performed showing no ova, cyst, or parasites. On the fifth day, the patient presented overnight with shortness of breath, tachypnea, and an oxygen saturation rate of 93-96%, needing a 2 L nasal cannula. That same night, the hepatic drainage was less than 30 cc with an improvement in the abdominal pain. An ameba antibody test was sent, with a negative result followed by an equivocal result two days later (Table 2).

**Table 2:** Enzyme immunoassay (EIA) antibody amebic test results

Date performed	06/08/22	06/10/22	06/15/22
Result	Negative	Equivocal	Positive

**Figure 2:**
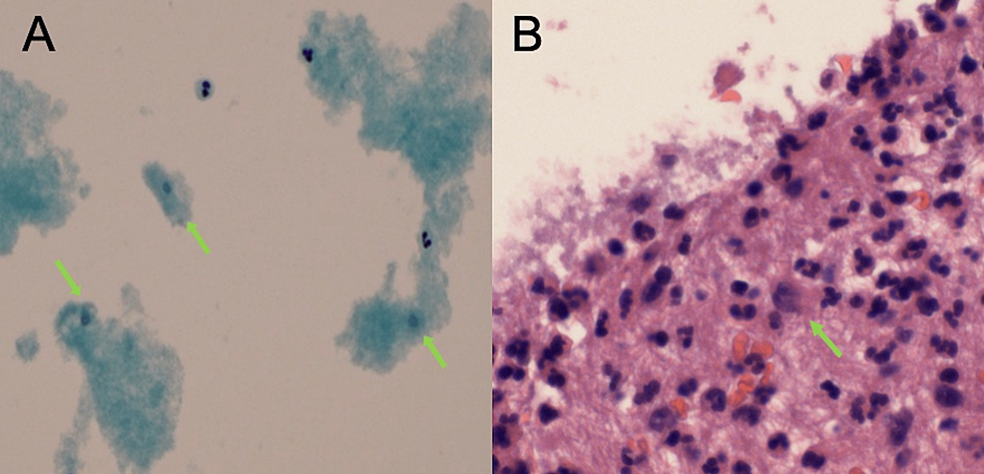
Pathology microscope images of pleural fluid: (A) Cell block, (H&E, x40): rare ill-preserved trophozoites (green arrow), along with abundant neutrophils and mild reactive mesothelial cells; (B) Thin prep cytology, (60X): possible occasional (spores) corresponding with amebiasis (green arrow). H&E: hematoxylin and eosin stain

Due to pleural effusion, seven days after admission, an ultrasound-guided right thoracentesis was carried out. During that procedure, 400 cc of yellow-cloudy fluid was obtained and sent for analysis. The pathology laboratory received the pleural fluid for the preparation of a cell block and cytology, and found rare ill-preserved trophozoites in the cell block section along with abundant neutrophils and mild reactive mesothelial cells (Figure 2).

The thin prep cytology showed possible occasional (spores) corresponding with amebiasis (Figure 2). Despite the equivocal result in the last antibody test, a confirmation/additional testing for ameba was suggested due to the high possibility of a false negative result in the first weeks of the disease. A hepatic fluid culture was repeated after seven days, with a positive result for alpha streptococci; consequently, metronidazole was removed from the current regimen.

The patient underwent a CT scan, reporting a slight decrease in the size of each of the abscesses with pigtail catheters and no significant change in the smallest abscess. A small amount of fluid was seen lateral to the liver, minimal decrease in the loculated pleural effusion and mild splenomegaly. Even though imaging results improved, the patient’s clinical condition continue to deteriorate, exhibiting spiking fever, tachycardia, tachypnea, and increased abdominal pain.

By day 14, a third ameba antibody confirmation/additional test was positive (Table 2), clarifying the etiology of both, the liver abscesses and the pleural effusion. Therefore, metronidazole was reestablished in the patient’s treatment plan. Five days after metronidazole was reinitiated, a CT scan was performed showing no abnormalities in the liver and lungs. Due to this result, a hepatic drainage check was also performed reporting no significant residual cavities, leading to the removal of the drainage catheters. Laboratory analysis showed an improvement of 4,000 k/mm^3^ in the WBC count. After 26 days of hospitalization, the patient displayed significant clinical and radiological improvement. The patient was discharged with a home treatment plan that consisted of amoxicillin 500 mg and metronidazole 500 mg, orally every 12 hours.

**Figure 3:**
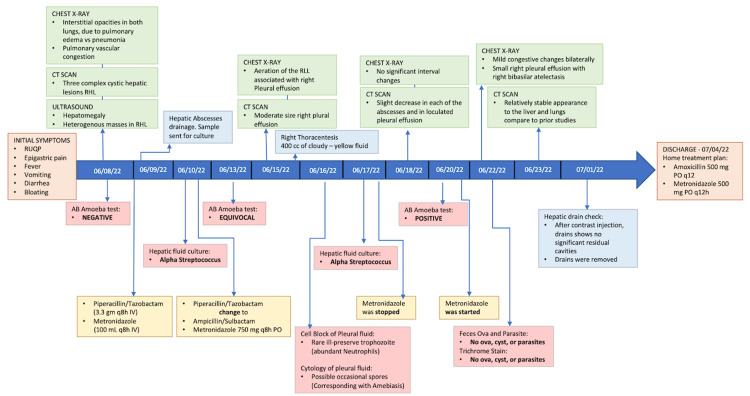
Timeline of disease progression CT, computed tomography; AB, antibody; RUQP, right upper quadrant pain; RHL, right hepatic lobe; RLL, right lower lobe; IV, intravenous; PO, enteral; q, quaque; h, hour; mg, miligrams; cc, cubic centimeter

The patient’s disease progress is illustrated as a timeline(Figure 3)*.*

## Discussion

Approximately 12% of the world's population is affected by amebiasis. It is a benign condition with occasionally no clinical symptoms, in contrast to intestinal invasive disease, which causes weight loss, watery diarrhea, and discomfort in the abdomen [7].

*E. histolytica* has two life stages: the cystic stage, which is the infectious stage, and the trophozoite stage, which results in the development of invasive disease. After the ingestion of the quadrinucleate cyst, excystation in the small intestinal lumen is followed by the production of motile, potentially invasive trophozoites [8]. Cysts can remain viable in the environment for weeks to months; the ingestion of a single cyst is sufficient to cause disease. The trophozoite has the ability to kill both, epithelial cells and inflammatory cells. This is thought to occur through the secretion of proteinases by the trophozoites, lysis of target cells via a contact-dependent mechanism. Another mechanism of trophozoite invasion is the killing of mammalian cells by apoptosis.The ability to form amebapores in lipid bilayers results in cytolysis of infected cells. Additionally, the intestinal permeability is altered by the trophozoite, most likely by the disruption of proteins in the tight-junction [4]. The spread of the trophozoites to extraintestinal locations like the liver (by hematogenous spread through the portal circulation) and peritoneum can happen after they infiltrate the colonic epithelium [9]. The organism causes hepatic inflammation, which is followed by necrosis, and this leads to the formation of an abscess [10]. One of the most frequent consequences is an amebic liver abscess, which affects 3-9% of patients and causes significant morbidity and mortality [7]. The typical patient with an amebic liver abscess in the United States is an immigrant from an endemic area, a man aged 20-40 years with fever, right upper quadrant pain, leukocytosis (>10.000 cells/μL), abnormal serum transaminase and alkaline phosphatase levels, and a defect seen on hepatic imaging studies [2]. Complications of amebic liver abscess (ALA) involve rupture of the abscess causing it to spread into the peritoneum, pleural space, or pericardium [10].

The infection usually spreads to the lungs by extension of an amebic liver abscess and may pass to the thorax directly from the primarily intestinal lesion through hematogenous spread; however, inhalation of dust-containing cysts of *E. histolytica* is another possible route of direct infection in the lung without a liver lesion [11]. Unilateral pleural effusion was a frequent finding on chest x-ray, occurring in 20% of the patients [9]. An essential tool for a healthcare professional to identify potentially harmful living organisms in the bloodstream is blood culture. However, the usefulness of this crucial tool may be constrained by false-positive findings. When organisms that are not actually present in a blood sample are grown in culture, contamination occurs, which causes false positives in blood culture [12].

Serum antigen detection has a sensitivity of over 95% with serologic testing (indirect hemagglutination) having a sensitivity of 70-80% in acute disease and greater than 90% in the convalescent state. It should be noted that in the first week of the disease course, there may be false-negative serologic tests. On the other hand, stool microscopy has a sensitivity of only 10-40% [8]. Therefore, rapid serodiagnosis in patients suspected of amebic abscess is often an important tool in clinical decision-making and can be of help in the reduction of the costs of additional treatment and prolonged hospital stay [6]. 

The preferred medication for amebic liver abscesses is still metronidazole. Amebicides have considerably reduced disease-related mortality and morbidity during treatment [7]. Occasionally, in addition to antiparasitic treatment, an amebic liver abscess requires to be aspirated for therapeutic purposes. Patients with a high risk of abscess rupture, as indicated by a cavity with a diameter of more than 5 cm or by the presence of lesions in the left lobe, should be given the option of having the abscess drained if there has not been clinical improvement after five to seven days of medication therapy [5].

## Conclusions

Even though the prevalence of amebiasis is low in the United States, we need to be able to recognize this disease to prevent future complications, such as ALA. This report was of a 35-year-old patient who arrived at the emergency room, exhibiting the classic clinical/radiologic scenario of amebiasis, along with the typical epidemiological components including, sex, age range, and geographic location. All of these characteristics are essential to recognize the disease and provide adequate treatment, in order to contribute to the patient’s successful recovery.
